# Attitudes towards generative-AI based Virtual patient systems in crisis support training

**DOI:** 10.1192/j.eurpsy.2025.216

**Published:** 2025-08-26

**Authors:** S. K. Mårtensson

**Affiliations:** IHV, Institution of health science, Skövde, Sweden

## Abstract

**Abstract:**

While generative AI (genAI) has made significant advances, millions of people are facing humanitarian crises, resulting in the denial of their basic human rights. One humanitarian response to addressing humanitarian crises is crisis support teams with knowledge of psychological first aid (PFA). In humanitarian crises, skilled practice in PFA by crisis support teams can strengthen the mental health of affected individuals, which can be crucial to ensuring societal well-being. At the same time, there are major challenges in training crisis support teams in PFA. With advancements in genAI, there are opportunities to develop virtual patient systems to enhance PFA training for crisis support teams. This presentation will share preliminary data collected through the genAI-agents survey, which explores technological openness, attitudes and learning through genAI and genAI-based virtual patient systems in healthcare education as well as specifically targeted questions to the genAI-based VP system Crisis Support-VR. This survey is a part of a larger project focusing a central research question Does Crisis Support-VR enhance the skills and ability of crisis support teams to deliver effective PFA, thereby strengthening the mental health of individuals affected by humanitarian crises with two interconnected sub-goals; • to empirically explore health and medical staff within children, youth and adult services learning in Crisis Support-VR, • to develop an educational module for training and practice opportunities in PFA to support and help national and international organizations train crisis support teams in applying PFA to children, youths an adults affected by humanitarian crises.

**Disclosure of Interest:**

None Declared

